# De Novo Ion-Exchange Membranes Based on Nanofibers

**DOI:** 10.3390/membranes11090652

**Published:** 2021-08-25

**Authors:** Shaoling Zhang, Akihiko Tanioka, Hidetoshi Matsumoto

**Affiliations:** 1 Department of Materials Science and Engineering, Tokyo Institute of Technology, 2-12-1 Ookayama, Meguro-ku, Tokyo 152-8552, Japan; 2 Interdisciplinary Cluster for Cutting Edge Research, Institute of Carbon Science and Technology, Shinshu University, 4-17-1, Wakasato, Nagano 380-8553, Japan; atani417@j03.itscom.net

**Keywords:** ion-exchange, nanofiber, composite, membrane, catalyst, separation, adsorption, fuel cell

## Abstract

The unique functions of nanofibers (NFs) are based on their nanoscale cross-section, high specific surface area, and high molecular orientation, and/or their confined polymer chains inside the fibers. The introduction of ion-exchange (IEX) groups on the surface and/or inside the NFs provides de novo ion-exchangers. In particular, the combination of large surface areas and ionizable groups in the IEX-NFs improves their performance through indices such as extremely rapid ion-exchange kinetics and high ion-exchange capacities. In reality, the membranes based on ion-exchange NFs exhibit superior properties such as high catalytic efficiency, high ion-exchange and adsorption capacities, and high ionic conductivities. The present review highlights the fundamental aspects of IEX-NFs (i.e., their unique size-dependent properties), scalable production methods, and the recent advancements in their applications in catalysis, separation/adsorption processes, and fuel cells, as well as the future perspectives and endeavors of NF-based IEMs.

## 1. Introduction

Ion-exchange membranes (IEMs) comprise immobilized ionizable functional groups and oppositely charged ions that selectively exchange target ions. IEMs are typically composed of inert substrates, immobilized charge groups, and exchangeable counter-ions. Based on the type of immobilized charge groups, IEMs are broadly classified into cation-exchange membranes (CEMs) and anion-exchange membranes (AEMs). CEMs contain fixed negative-charge groups such as sulfonic acid, phosphonic acid, and carboxylic acid groups, accompanied by positive-charge counter-ions. AEMs contain fixed positive-charge groups and exchangeable anionic counter-ions, and the most common positive-charge groups are quaternary ammonium cations, imidazole cations, and guanidinium cations. In addition to common CEMs and AEMs, there are a few special types of IEMs such as proton-exchange membranes (PEMs), bipolar membranes (BPMs), and amphoteric membranes. PEMs are a common type of CEMs and are mainly used to conduct protons in fuel cells. BPMs contains a cation-exchange layer and an anion-exchange layer. They are mainly used for electrodialysis (ED) in which water will dissociate into hydrogen ions and hydroxide ions at the interface of the two layers. Amphoteric membranes contain both cation and anion-exchange groups [1,2,3].

Currently, IEMs are commercialized and widely used in diverse fields such as water treatment, ultrapure water production for the semiconductor industry, catalytic conversion processing, flow battery/fuel cell technologies, pharmaceutical processing, and biotechnology. The most desired properties for IEMs are high permselectivity and low electrical resistance, in addition to good mechanical stability [4,5].

Nanotechnology has experienced rapid growth over the last two decades with expanding research interest in the unique properties of nanomaterials. Nanofibers (NFs) are 1D nanomaterials with a nanoscale diameter and macroscale length. NFs exhibit promising functionalities and ease of manipulation due to their nanoscale structure and macroscopic length, respectively. NFs offer unique physical, mechanical, and electrochemical properties, and thus evolved as an exciting new class of materials for membranes used in various fields including energy, environment, and health [6,7,8,9].

**Figure 1:**
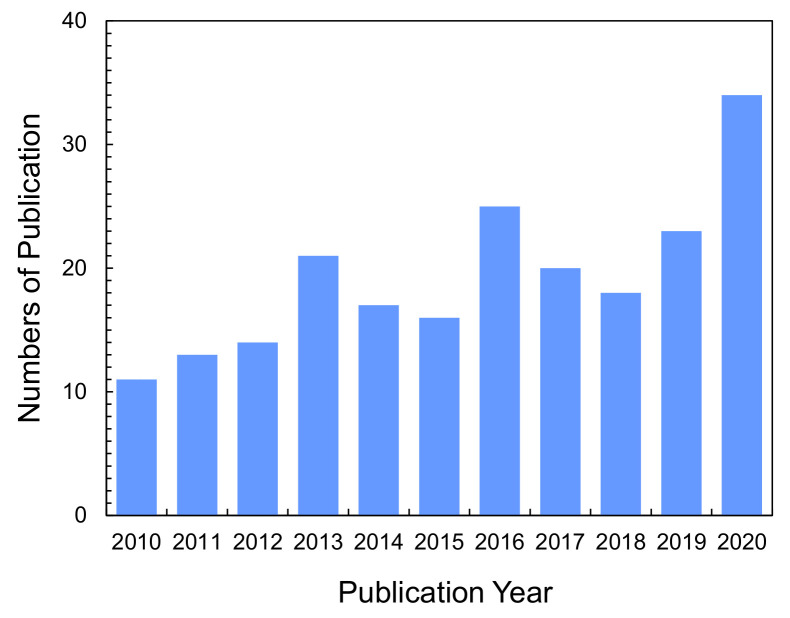
Numbers of publications on ion-exchange nanofibers. Graph showing the research articles and letters published in recent years on “ion-exchange nanofibers” topics. These numbers were obtained from SciFinder^®^ in July, 2021.

**Figure 2:**
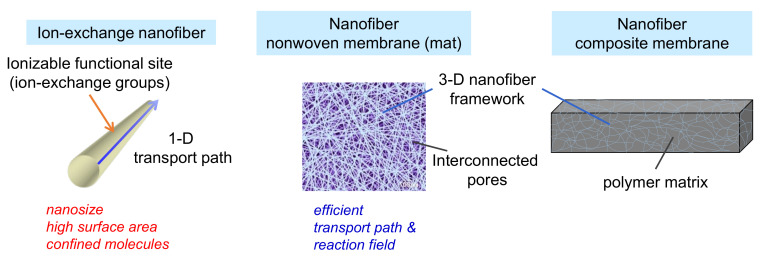
Characteristics of ion-exchange nanofibers, porous nanofiber non-woven membranes (mat), and dense nanofiber composite membranes.

Recently, ion-exchange NFs (IEX-NFs) have attracted considerable attention. As shown in Figure 1, the number of publications dealing with IEX-NFs has grown steadily. The combination of ionizable groups and NFs improves the performance of IEMs. Figure 2 shows the characteristics of IEX-NFs. IEX-NFs exhibit high catalytic efficiency, high ion-exchange and adsorption capacities, and high ionic conductivity due to their size-dependent features, such as large surface area, more available functional groups, molecular orientation, and/or confined polymer chains [8]. Furthermore, an IEX-NF network is a promising option for nanoscale morphology control and molecular ordering in a polymer matrix. Thus, IEX-NFs expand the scope of IEMs from traditional dense membranes to de novo highly porous ones and their dense composite ones. In particular, their large surface areas significantly improve the catalytic activity and adsorption capacity beyond the limitations due to the very low surface area of traditional dense IEMs. We have already reported the first review paper in this field in 2018 [9]. The review, however, did not cover the applications such as catalysis and the scalable NFs such as solution blown NFs and nanofibrillated aramid fibers.

The present review highlights the fundamental aspects of IEX-NFs including their unique size-dependent properties and scalable production methods such as electrospinning, solution blow spinning, and nanofibrillation. Furthermore, we summarize the more recent advances in the application of IEMs, such as catalysis, membrane separation/adsorption, and fuel cells, and discuss the future perspectives and endeavors of NF-based IEMs.

## 2. Fundamental Aspects and Production Methods of Ion-Exchange Nanofibers

**Figure 3:**
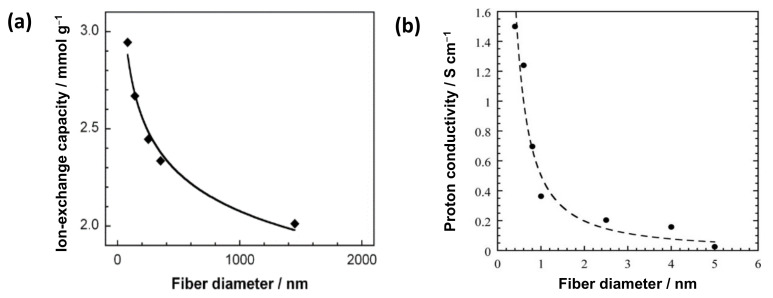
Effect of nanosize on the electrochemical properties of IEX-NFs. (**a**) Ion-exchange capacity vs. fiber diameter of surface sulfonated carbon NFs with fixed surface charge density [10]. (**b**) Proton conductivity (at 30 °C, 90% RH) vs. fiber diameter for high-purity Nafion NFs [11].

The reported nanosize effects on the electrochemical properties of IEX-NFs are shown in Figure 3. Imaizumi et al. reported a typical trend of nanosize effect on the ion-exchange capacity (IEC) of surface sulfonated carbon NFs with a constant surface charge density (Figure 3a) [10]. The specific surface area increases with a decrease in the fiber diameter, thereby improving the amount of ionizable groups on the NF surface. In addition, Dong et al. reported the influence of nano-dimensions on the ionic conductivity of IEX-NFs. The proton conductivity of Nafion^®^ (Chemours, Wilmington, DE, USA) NFs sharply increases with the reduction of fiber diameter to nanometer (400 nm NFs exhibit a maximum conductivity of 1.5 S·cm^−1^, higher than the bulk film 0.1 S·cm^−1^, as shown in Figure 3b) [11]. The improved conductivity is ascribed to the orientation of ionic domains along the Nafion NF axis. Thus, an increase in specific surface area and molecular orientation improves the properties of ion-exchangers. 

**Figure 4:**
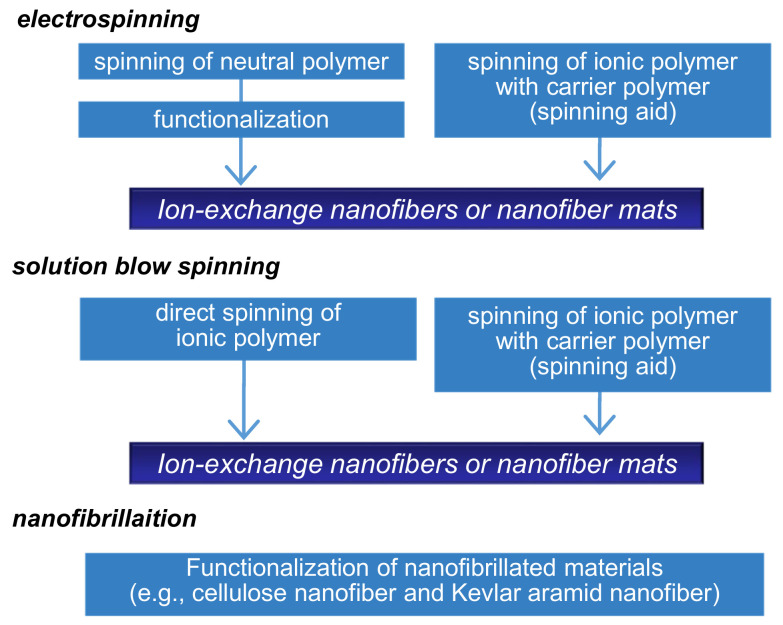
Production scheme of ion-exchange nanofibers.

There are several methods to prepare NFs including solution spinning, melt spinning, molecular assembly, and chemical vapor deposition. Electrospinning is one of the most versatile approaches based on electrohydrodynamic principles and is used for the one-step formation of NF non-woven membranes (or mats) [6,7,8,12,13,14]. Diverse polymeric formulations, such as polymer solutions in solvents, molten polymers, and polymer solutions with inorganic sols, can be electrospun. A typical electrospinning setup consists of three major components: a high-voltage power supply, a spinneret, and a grounded or oppositely charged collector. When an external electric field is applied to the spinning solution that is fed through the spinneret, a suspended conical droplet is formed at the tip of the spinneret. Furthermore, with an increase in the electric field, the electrostatic force overcomes the surface tension of the polymer solution, ejecting a tiny jet from the tip of the conical droplet and is drawn towards the collector. Most of the solvent then evaporates before arriving at the collector and NF non-woven membranes are formed on the collector surface. The fiber morphology and diameter can be controlled by modulating the solution properties (e.g., viscosity, electrical conductivity, and boiling point), operating parameters (e.g., applied voltage and distance between the spinneret and collector), and ambient conditions (e.g., temperature and humidity). The IEX-NFs can be electrospun in two ways, as shown in Figure 4. First, IEX-NFs are directly electrospun from natural, synthetic polyelectrolytes [13,14,15,16,17] and polyelectrolyte complexes [18,19,20]. However, electrospinning of polyelectrolytes is often challenging because inherent electrical charges (i.e., ion-exchange groups) can cause instability of the polymer jet under high electric fields. In such cases, it becomes necessary to add small amounts of spinning aids (e.g., a high-molecular-weight hydrophilic polymer or inorganic sol) or to use alternative solvent (e.g., ethanol/KOH) [21]. Second, electrospun neutral NFs are functionalized to obtain IEX-NFs [22,23,24,25,26]. For example, Matsumoto et al. initially electrospun respective solutions of poly(styrene) (PS) and poly(4-vinylpyridine) (P4VP) solutions, followed by sulfonation of PS mats and quaternization of P4VP mats to obtain cation and anion-exchange membranes [23]. The IEC of the functionalized NF membranes ranged from 0.78 to 1.34 mmol·g^−1^. Similarly, a wide variety of IEX-NFs are successfully prepared through electrospinning. 

Low production of NFs is a critical challenge for the practical application of IEX-NFs. Therefore, several research groups have attempted scaling-up NF production using methods such as free-surface electrospinning, multi-nozzle electrospinning, solution blow spinning (SBS), and centrifugal spinning [27]. In particular, SBS has several merits over other processes, as it is simple, safe (no requirement for high electric field), highly efficient, amenable to a wider range of solvents, and offers precise control over fiber structure and diameter [28]. SBS involves high-speed stretching of airflow and employs the Bernoulli principle, where a change in air pressure transforms to the kinetic energy of the spinning solution. Shinkawa et al. reported the preparation of high-purity perfluorinated sulfonic acid (Nafion^®^) NFs via SBS [29].

In contrast, Yang et al. developed alternative organic NFs based on aramid. Aramid fibers contain highly aligned molecular chains of poly(paraphenylene terephthalamide) (PPTA). PPTA forms strong intermolecular bonds through dense hydrogen bonds and π−π stacking, resulting in an elastic modulus and strength comparable to carbon fibers. Kevlar^®^ aramid nanofibers (KANFs) were fabricated by splitting the commercial Kevlar^®^ fabrics (Du Pont, Wilmington, DE, USA) and could be functionalized with desired chemical groups. KANFs are potential platforms for high-functional IEX-NF due to their special properties of electrical insulation, flexibility, mechanical robustness, physical stability, and organic solvent resistance [30].

As shown in Figure 2**,** the NF-based IEX membranes are generally divided in two categories: porous NF non-woven membranes and dense NF composite membranes. The former type can be directly obtained from electrospinning or SBS. For applications such as dialysis membranes and BPMs, a hot-pressing process is necessary to obtain dense membranes [31]. In addition, the KANF-based IEMs are usually prepared by casting Kevlar NF solutions, which are obtained by dissolution of Kevlar aramid fiber [32,33,34]. The dense NF composite membranes are usually prepared by casting polymer solutions on NF networks, followed by a drying process [35].

The porous NF non-woven membranes have several prominent properties such as a large specific surface area, high porosity, uniform nanoscale/microscale pore size distribution, and low tortuosity due to the interconnected pores formed between the fibers [8,36]. These unique features are vital for their applications as catalyst and separation/adsorption membranes. In addition, IEX NF networks provide a promising option for nanoscale morphology control and molecular ordering in a polymer matrix. As an example, three-dimensional (3D) randomly oriented NF networks enabled the construction of a continuous transport pathway in a polymer matrix [35]. Characteristics of traditional IEMs and IEX-NF non-woven membranes are summarized in Table 1. 

## 3. Applications of Nanofiber-Based IEMs 

The IEX-NFs can be directly applied as porous NF membranes and/or composites of porous NF frameworks within polymer matrices (dense NF composite IEMs). This section focused on the application of NF-based IEMs in catalysis, membrane separation/adsorption, and fuel cells. 

### 3.1. Catalysis

NF non-woven membranes (NF mats) have several merits such as a large specific surface area and pore volume, controllable pore size distribution, and negligible resistance to intra-membrane mass transfer. Furthermore, NF membranes enable easy recovery and recycling of expensive catalysts and enzymes. Hence, NF mats hold great promise as both a catalyst and as providing catalyst support. Several reports employ NF mats as support for catalyst nanoparticles [42,43] and enzymes [44]. However, this section exclusively focuses on IEX-NF mats used as catalysts.

**Figure 5:**
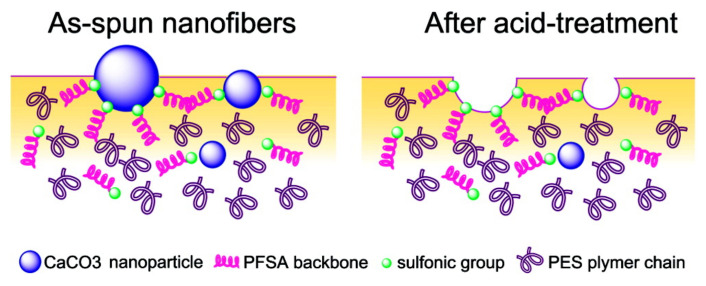
Surface schematic of the PES/PFSA NF containing CaCO_3_ nanoparticles before (left) and after (right) acid treatment [49].

Perfluorosulfonic acid (PFSA) resins are used as catalysts for several reactions. However, the low surface area of the resin (less than 0.02 m^2^·g^−1^ [45]) implies that a majority of sulfonic acid groups, serving as active sites, remain buried in the resin matrix, limiting the catalytic activity. Therefore, enhancing the availability of surface functional groups by increasing the surface area of PFSA is an efficient way to improve its catalytic activity. The preparation of PFSA NFs is one method for increasing the specific surface area. Chang et al. [46] attempted to electrospin a PFSA/poly(vinyl alcohol) (PVA) blend solution to prepare well-formed NF mats. The mats were used for catalyzing the esterification of ethanol and acetic acid. The PFSA/PVA NF mats exhibited good catalytic activity in ethyl acetate synthesis, with an efficiency proportional to the specific surface area of the NFs mats. The addition of the non-solvent to the electrospinning solution further improved the utilization of sulfonic acid groups in PFSA molecules [47]. Moreover, addition of nanoparticles [48,49] also improved the density of surface acid sites. Lu et al. fabricated a poly(ether sulfone) (PES)/PFSA/SiO_2_ NF mat with a high surface area (85.6 m^2^·g^−1^) [48]. The prepared membrane was excellent at catalyzing the esterification of ethanol and acetic acid, and it exhibited satisfactory durability and reusability. Lu et al. also reported the incorporation of CaCO_3_ nanoparticles in the PES/PFSA NF mats [49]. Acid treatment enhances the content of sulfonic groups on the external surface (Figure 5), thus making the reaction sites accessible to attachment by ethanol and acetic acid. Furthermore, the prepared PES/PFSA NF mats exhibit acceptable catalytic performance in esterification reactions. The maximum conversion rate of acetic acid reached 68% within 8 h and is comparable to conventional catalysts (98% H_2_SO_4_, 75% acetic acid). Moreover, the prepared mat allows for easy separation and recovery, making it a potential substitute for traditional liquid acid catalysts. In addition to PFSA, NF mats of phosphotungstic acid (PWA)/PVA were fabricated as catalysts for biodiesel production [50]. Shi et al. investigated the effect of PVA concentration and PWA content on the NF structure and catalytic performance. They found that 12 wt.% PVA and 20 wt.% PWA resulted in smooth and homogenous NFs with an average fiber diameter of 157.2 nm and BET surface area of 66.76 m^2^·g^−1^. The PWA/PVA mats exhibited a reaction rate constant of 2.647 min^−1^ with an effectiveness factor of 0.92 min^−1^, superior to the commercial IEX resin NKC-9. NKC-9 is a cation-exchange resin comprised of copolymer of styrene and divinyl benzene, containing sulfonic acid groups, with ion-exchange capacity of 4.7 mmol·g^−1^, surface area of 77 m^2^·g^−1^, and average pore diameter of 56 nm. Furthermore, the prepared mat showed excellent catalytic stability and maintained a stable conversion over 10 days. 

Kumar et al. [51] reported the application of carbon nanofibers (CNFs) as a metal-free and non-precious element catalyst for reducing carbon dioxide (CO_2_) to carbon monoxide (CO). The CNFs were prepared by carbonizing the electrospun poly(acrylonitrile) (PAN) NFs at 1050 °C in an argon environment. The prepared CNFs exhibited a current density ~13 times higher than the bulk silver catalyst for the selective conversion of CO_2_ to CO. The excellent catalytic proficiency of CNFs was attributed to the positive charges on carbon atoms due to the existence of nitrogen atoms within the carbon lattice. Moreover, the nanofibrous structure provided a large number of active sites. 

### 3.2. Membrane Separation

Advantages of porous NF non-woven mats concern its high specific surface area, high porosity, uniform nanoscale or microscale pore size distribution, and low tortuosity. These features are inevitable for their application as separation membranes [52,53]. 

IEX-NF mats find use in ionic separation processes such as diffusion dialysis (DD) and electrodialysis (ED). Pan et al. prepared a CEM of electrospun sulfonated PPO NFs for application in DD [54]. During the alkali recovery from Na_2_WO_4_/NaOH solution, the CEMs showed a higher hydroxide permeability (9.7 mm^2^·h^−1^) and separation factor (36.1) than solution-cast membranes (6.1 mm^2^·h^−1^ and 21.8, respectively). In addition, Zheng et al. prepared PVDF NF mats by multi-jet electrospinning arranged in an arc array with sheath gas. The mats were treated with 98% concentrated sulfuric acid to add reactive exchange groups, followed by a hot-pressing process. ED using the prepared membranes demonstrated a desalination ratio of >50% within 30 min for the NaCl solution [31]. 

**Figure 6:**
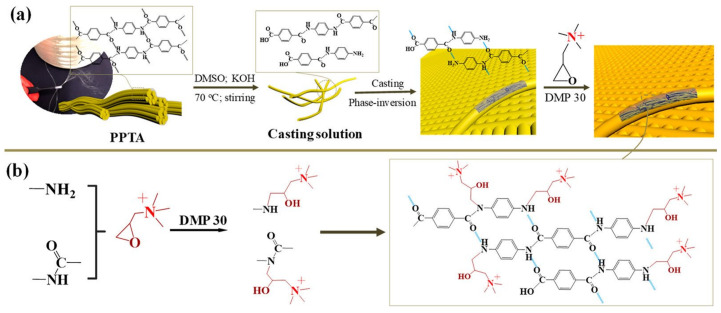
(**a**) Process and (**b**) mechanism of fabricating ANF#QA membranes [32].

Kevlar-based functional PPTA NFs are drawing attention to the preparation of novel IEMs. Zhao et al. fabricated high-performance AEMs by dissolving PPTA NFs followed by a reaction with quaternary ammonium groups (Figure 6). The prepared aramid NF quaternary ammonium membranes (ANF#QA) reached a high IEC of 1.75 mmol·g^−1^. Furthermore, the membranes exhibited a high desalination and concentration efficiency with selective separation of Cl^-^/SO_4_^2-^ during ED [32]. Shen et al. fabricated a series of organic solvent-resistant CEMs by splitting Kevlar fabrics into small and short NFs, followed by modification with 4-amino-benzenesulfonic acid monosodium salt and poly(4-styrenesulfonic acid-co-maleic acid) sodium salt (PSSMA) via amide condensation. The prepared CEMs showed a high IEC of up to 2.23 mmol·g^−1^ with a low surface area resistance of 2.40 Ω·cm^2^. Moreover, ED with an optimized membrane composition revealed high desalination efficiency and concentration efficiency in organic solvents [33]. Zhao et al. designed a CEM by interpenetrating networks of PSSMA into KANFs, followed by functionalization with 4-amino-2,2,6,6-tetramethylpiperidine-1-oxyl. The prepared membrane had a thickness of ~8 µm and exhibited a high membrane limiting current density of 32.0 mA·cm^−2^ (in 0.1 M NaCl solution) with an exceptional desalination efficiency (99.9% for NaCl) in ED. Moreover, the separation of Li^+^/Mg^2+^ with the prepared membrane is comparable to that of commercial CEMs with monovalent selectivity [34]. Zhao et al. reported a KANF-based CEM with a high performance for ion separation in ED with a maximum desalination efficiency of 99.7% after 220 min for Na_2_SO_4_ and significant selectivity for monovalent cations. Moreover, KANF-based membranes demonstrate exceptional desalination at high temperatures (as high as 100 °C) and organic solvent/aqueous environments (as high as 80% acetone solution) [55]. 

Additionally, some studies reported the application of IEX-NFs as a composite along with BPMs. Wakamatsu et al. prepared anion-exchange NF mats by electrospinning poly(4-vinylpyridine) followed by quaternization [56]. They investigated the effect of IEX-NF mats as an intermediate layer in the BPM composite for water splitting. The prepared BPM improved with accelerated water dissociation. Pan et al. prepared a sandwiched BPM by consequent electrospinning of the respective layers of sulfonated PPO, poly(ethylene glycol), and quaternized PPO [57]. The prepared BPM showed an extremely low potential drop compared to the corresponding cast membrane. Shen et al. reported the preparation of a BPM with a high-interfacial area three-dimensional (3D) water-splitting junction [58]. The inner bipolar junction comprised of interpenetrating anion-exchange (quaternized PPO) and cation-exchange (sulfonated poly(ether ether ketone)) NFs enabled water splitting at ultrahigh current densities of up to 1.1 A·cm^−2^ without membrane dehydration or blistering. Furthermore, Hohenadel et al. prepared BPMs with 3D dual-fiber junctions by electrospinning cation and anion-exchange polymers simultaneously. The high interfacial surface area was important for both efficiency of water dissociation and membrane selectivity [59]. 

Bacterial fouling in pressure-driven membrane processes may decrease the water flux and deteriorate the water quality. Therefore, membrane materials with antibacterial activity are valuable for application. The IEX-NFs also improve the antibacterial properties of membranes. Cheah et al. prepared quaternized chitosan NFs from electrospun PAN NFs by hydrolyzation, covalently grafting chitosan molecules, followed by functionalization with quaternary amine. The prepared membranes exhibited an antibacterial activity of up to 99.95% against *E. coli.* [60]. He et al. prepared an active PAN/La(OH)_3_ NF web through the combination of electrospinning and subsequent in situ alkaline treatment. The prepared membrane showed a large water flux at low pressure and high bacterial rejection. Furthermore, the NF composite membrane exhibited high efficiency and rapid phosphate removal because of the active binding sites for phosphate generated by the well-dispersed La(OH)_3_ nanorods in PAN NFs. The membrane has potential applications in microfiltration to remove cells and reduce contamination [61]. Chen et al. mixed fluxible poly(*p*-phenylene terephthalamide) (f-PPTA) with PVDF to obtain a hydrophilic NF membrane through electrospinning. The f-PPTA acted as a water channel in the membrane, accelerating the flow of water molecules through the membrane. The as-prepared membrane exhibited excellent antibacterial behavior against *E.coli* [62].

### 3.3. Membrane Adsorption

IEX NF membranes have competitive structural advantages including a large specific surface area, high porosity, good pore connectivity, controllable single fiber, and assembly structure. Membrane adsorption involves two processes: static and dynamic. For static adsorption, the membrane acts as an adsorbent and its surface area is the primary factor that determines the adsorption capability. For dynamic adsorption, the membrane performs the functions of both adsorption and filtration with a single-pass flow. IEX-NF membranes exhibit greater adsorption in less time [63] due to the abundant surface functional groups. Hence, IEX-NF membranes are promising candidates for dye and heavy metal removal as well as for protein separation/purification [64,65,66,67]. 

#### 3.3.1. Anion-Exchange Nanofiber Membranes

Porous NF non-woven membranes with tertiary amine, quaternary ammonium, or other positively charged groups were investigated for use as anion-exchange adsorbents. Chitosan is extensively used as an adsorbent because of its low cost, non-toxicity, and high content of amino and hydroxyl functional groups. Chitosan-based NFs are extensively researched for removal of metal ions such as As^5+^ [68], Cr^6+^ [69], and Cu^2+^ [70]. For example, Min et al. [68] investigated the adsorptive removal of arsenate using chitosan NFs. A higher adsorption capacity for As^5+^ was obtained at low pH and the adsorption equilibrium was achieved in 0.5 h. The maximum adsorption capacity was 30.8 mg·g^−1^, higher than most of the reported chitosan adsorbents. In addition to chitosan, other positively charged NFs are being investigated as heavy metal adsorbents. Zeytuncu et al. prepared PVA/(maleic anhydride)/acryloyl thioamide monomer NF mats using a combination of electrospinning and UV irradiation [40]. The mats revealed a maximum adsorption of 69.93 and 112.36 mg·g^−1^ for Pt^4+^ and Pd^2+^ (as chloro-anionic complexes of PtCl_6_^2−^ and PdCl_4_^2−^ in a lower pH) at 45 °C and pH 1.1, respectively. Wang et al. prepared PAN/polypyrrole [71] and PAN/polyaniline [72] core-shell structured NFs by electrospinning of PAN combined with in situ polymerization of pyrrole and aniline monomers, respectively. The NF membrane exhibited excellent adsorption of Cr^6+^. Furthermore, Plíštil et al. reported the preparation of anion-exchange NFs by a two-step functionalization of PS NFs with chlorosulfonic acid and ethylenediamine [73]. The IEC of the membrane reached 4.0 mmol·g^−1^. 

Wastewater containing organic dyes is a serious ecological and public health risk. Anion-exchange NF membranes are used for dye decontamination from wastewater. Ma et al. developed composite NF membranes by electrospinning mixed solutions of methacrylated poly(ethylenimine) and PVDF [74]. The prepared membranes effectively removed methyl orange (MO) from an aqueous solution, with a maximum adsorption as high as 633 mg·g^−1^. The study confirmed that the charge properties of the adsorbent played an important role in dye adsorption. Song et al. prepared poly(ethyleneimine) NF membranes by electrospinning and crosslinking, and the membranes exhibited exceptional adsorption of heavy metal ions and MO. Moreover, the adsorption rate of MO remained at ~75% after four cycles, while the adsorption rate of copper and lead remained ~90% after five cycles [75]. Thus, the membrane allowed for repeated use. Notably, Chen et al. prepared a novel multifunctional electrospun cellulose acetate fiber membrane functionalized by deacetylation, carboxymethylation, and poly(dopamine) (PDA) coating. The prepared PDA@DCA-COOH membrane comprised of carboxyl, hydroxyl, and amine multifunctional groups exhibited maximum adsorption of 69.89 and 67.31 mg·g^−1^ for methylene blue and Congo red, respectively [76]. 

Additionally, anion-exchange NF membranes show potential for protein separation and purification. Matsumoto et al. reported that the adsorption behavior of DNA correlates well with electrokinetic properties of the electrospun chitosan NF membranes [38]. Rajesh et al. developed a cellulose-graft-poly(ethyleneamidoamine) anion-exchange NF membrane, exhibiting excellent static adsorption capacity (239 mg·g^−1^) for bovine serum albumin (BSA) [77]. Notably, Chen et al. grafted tertiary amine ligands onto electrospun poly(sulfone) (PSf) and PAN membranes using UV-initiated polymerization. Static and dynamic binding capacities for BSA were ~100 mg·mL^−1^ and ~200 mg·mL^−1^ for the functionalized PAN and PSf NF membranes, respectively [78]. Furthermore, Zhang et al. reported the preparation of adsorptive membranes made from electrospun cellulose NFs followed by surface functionalization with diethylaminoethyl anion-exchange ligand [79]. The prepared adsorptive membrane had a higher static binding capacity (40.0 mg·g^−1^) for BSA compared to commercial membranes. Turnbull et al. reported the recovery of viral vector adenovirus 5 using a cellulose NF ion-exchange adsorbent derivatized with QA ligands. A high infective recovery of >90% was obtained with a 29-fold productivity improvement over classical, beaded, and packed bed resin process [80]. Liu et al. applied PAN NF membranes functionalized with tris(hydroxylmethyl)aminomethane (P-Tris) in affinity membrane chromatography for lysozyme adsorption [81]. They demonstrated the effectiveness of the P-Tris NF membrane for the recovery of lysozyme from a complex chicken egg white solution. The characteristics of the reported anion-exchange NF membranes are summarized in Table 2.

#### 3.3.2. Cation-Exchange Nanofiber Membranes

Porous NF non-woven membranes with acidic groups find use as cation-exchange adsorbents. The NF membranes containing sulfonic acid groups are widely investigated. For example, Zhao et al. prepared electrospun PFSA/poly(*N*-vinylpyrrolidone) NF membranes. The membranes showed good removal of Cu^2+^ and Ca^2+^ ions because of more exposed functional groups [82]. Sulfonation of electrospun NF membranes of various polymers such as PS [83,84] and PES [85] to prepare cation-exchange NF membranes for adsorption are reported. Kwak et al. prepared IEX NF membranes by sulfonation of electrospun PES NFs and the maximum adsorption for ammonium ions reached 14.08 mg·g^−1^ [85]. In addition, NF membranes functionalized with phosphoric acid and carboxylic acid groups are studied as cation-exchange adsorbents. Xie et al. electrospun a phosphate-functionalized PVA/poly(acrylic acid) (PAA) (PVA/PAA-PO_4_) NF membrane, which showed a competitive uranium uptake of 277.78 mg·g^−1^ [86]. Tian et al. modified the surface of electrospun cellulose acetate NFs membranes with poly(methacrylic acid) and applied them for heavy metal adsorption from aqueous solutions [87]. The adsorption capacity increased with pH, with high adsorption selectivity for Hg^2+^. Chitpong et al. fabricated an IEX membrane by grafting poly(itaconic acid) onto cellulose NF membranes [88,89]. The NF membranes exhibited high adsorption capacity (exceeded 220 mg Cd^2+^·g^−1^) and rapid uptake (volumetric productivity of 0.55 mg Cd^2+^·g^−1^·min^−1^) of cadmium from polluted waters [90]. In addition, Ullah et al. reported a method to crosslink PVA NFs with glutaraldehyde to enhance its adsorption capacity against metal ions such as Cu^2+^ and Pb^2+^ [91]. Choi et al. prepared thiol-functionalized cellulose NF membranes by deacetylation of electrospun cellulose acetate NFs and subsequent esterification with a thiol precursor. The prepared membranes showed efficient removal of Cu^2+^, Cd^2+^, and Pb^2+^ ions with maximum adsorption capacities of 49.0, 45.9, and 22.0 mg·g^−1^, respectively [92].

There are reports on the application of cation-exchange NF membranes for dye adsorption. Ning et al. demonstrated that sulfonated PS NF membranes can adsorb cationic blue dyes from water and the adsorption capability increases with the number of sulfonic acid groups [93]. Yin et al. fabricated sulfonated PES NF membranes [94] exhibiting high permeation flux (320 L·m^−2^·h^−1^) and high retention (>99.0%) against 0.2 µm-particles, MB, and Pb^2+^. Xu et al. prepared cation-exchange NF membranes by electrospinning a blend of PES, acrylic acid, and methyl methacrylate copolymer. The prepared membranes exhibited a maximum adsorption capacity of 2257.88 mg·g^−1^ for methylene blue (MB). Moreover, the NF membranes exhibited excellent recyclability (81.45% of initial adsorption capacity after five cycles) and high filtration–purification efficiency (>99% at a high flux of 100 mL·min^−1^) [95]. Wang et al. prepared water-insoluble alginate-based membranes by crosslinking sodium alginate NFs with calcium chloride [96]. The prepared membranes exhibited good adsorption of MB (2230 mg·g^−1^), a short equilibrium time (50 min), and separated MB/MO with a high separation efficiency even after five cycles. 

**Figure 7:**
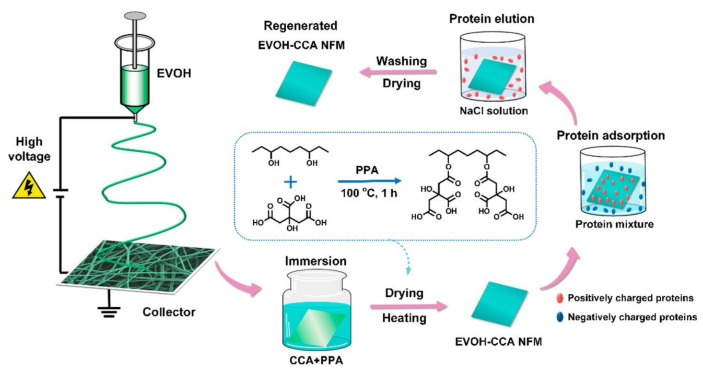
Fabrication process of EVOH-CCA NF membranes, their selective protein adsorption performance, and regeneration [39].

The abundant active carboxyl groups are useful for protein adsorption. Menkhaus et al. functionalized electrospun regenerated cellulose NF membranes through atom transfer radical polymerization of acrylic acid to create cation-exchange adsorption sites [97]. The prepared membranes showed better performance than the packed bed resins for static adsorption of lysozyme. Ding et al. fabricated highly carboxylated NFs by in situ graft polymerization of PVA NFs with maleic anhydride [98] or by functionalization of electrospun ethylene-vinyl alcohol (EVOH) NFs with critic acid (CCA) (Figure 7) [39]. Both membranes exhibited excellent integrated lysozyme adsorption. Figure 7 shows a scheme of the fabrication, selective protein adsorption, and regeneration of the EVOH-CCA NF membrane. Chiu et al. introduced –COOH groups to the PAN NF membranes through alkaline hydrolysis that demonstrated higher lysozyme adsorption than the commercial Sartobind^®^ C membrane (Sartorius AG, Goettingen, Germany) [37]. The prepared membranes were used for direct separation and purification lysozymes from chicken egg whites, improving the protein purification efficiency by 73.6 times [99]. Lee et al. used the membranes for purification of lysozymes from chicken egg white and achieved a high yield of 98% with a purification factor of 63 in a single step [100]. The characteristics of the cation-exchange NF membranes are summarized in Table 3.

#### 3.3.3. Hybrid Ion-Exchange Nanofiber Membranes

Porous hybrid NF non-woven membranes with IEX sites from inorganic/protein components are reported for adsorption. The inorganic nanoparticles possessing ion-exchange properties are attractive to be used for heavy metal removal and the NF carriers can circumvent aggregation and increase their active sites. There have been reports on the incorporation of metal oxide nanoparticles [101,102,103,104], zeolite nanoparticles [105], and hydroxyapatite nanoparticles (Hap NP) [106] in polymer NF membranes by electrospinning for heavy metal adsorption. For example, Tran et al. incorporated zeolite nanoparticles into electrospun cellulose acetate fibers to create free-standing IEX-NF membranes [107]. The zeolite nanoparticles embedded within the porous cellulose acetate fibers exhibited higher IEC than the free powders and the membrane showed improved selective ion exchange for Pb^2+^ over Cu^2+^. In addition, the high surface area of NF membranes can improve accessibility to the reactive sites and correspondingly enhance the adsorption efficiency. Jin et al. prepared a wool keratin/poly(ethylene terephthalate) composite NF membrane. The incorporation of keratin lead to improved hydrophilicity, large pore ratio, and extensive amino protonation. The prepared membrane displayed a maximum adsorption of 75.86 mg·g^−1^ for Cr^6+^ [108]. Xu et al. prepared electrospun mesoporous PAA/SiO_2_ composite NF membranes with the equilibrium adsorption for malachite green (MG) as high as 220.49 mg·g^−1^ [109]. The electrostatic interaction between the negative-charged silica surface and the positive-charged MG was considered to play an important role in the adsorption.

The synergetic effect between the incorporated component and host NF material is sometimes used for improving the removal efficiency. Tan et al. fabricated an adsorbent of lanthanum immobilized on electrospun chitosan NF (CS-NF). The arsenate adsorption of the prepared membrane was dependent on pH and reached up to 83.6 mg·g^−1^. The exceptional performance was ascribed to the structural characteristics: the host material CS-NF favored the pre-concentration of arsenate through electrostatic attraction and the immobilized lanthanum exhibited preferable arsenate removal through specific interactions [110]. Li et al. synthesized activated carbon (AC)-hybridized and amine-modified PAN NFs by blend electrospinning of PAN and AC, followed by amination treatment. The obtained amine-rich porous PAN NFs (APAN/AC) exhibited ultrahigh adsorption capacity for metal ions and dyes; the adsorption capacities for Cr^6+^ and MO were 284 and 248 mg·g^−1^, respectively. The exceptional adsorption capacity, significantly improved adsorption rate, and excellent recyclability were attributed to the synergistic effect of electrostatic attraction and extra storage space of AC [111]. The characteristics of the hybrid ion-exchange NF membranes are summarized in Table 4.

### 3.4. Fuel Cells

Polymer electrolyte fuel cells (PEFCs) are one of the most attractive electrochemical power converters with a wide variety of applications such as automotive power, stationary power, and microelectronics. The polymer electrolyte membrane in PEFCs is a crucial component for the selective transfer of protons from the anode to the cathode. In general, membranes with outstanding proton transfer abilities, low fuel penetrability, and high mechanical strength ensure the safe and stable operation of fuel cells, in addition to the high power density. There is growing interest to use ion-conductive NF composite membranes for improving PEFC performance [112,113]. Note that the NF composite membrane is a dense membrane rather than a porous one. The use of ion-conductive NFs as a 3D framework of composite polymer electrolyte membranes could break the conventional trade-off relationship between the ionic conductivity and mechanical properties of the membranes [114,115,116]. Sadrjahani et al. found that the proton conductivity of electrospun NF membranes of sulfonated poly(ether ether ketone) increased with the degree of sulfonation [117]. Thus, the ionic conductivity may improve by modulating the IEC. However, increasing the charge groups often results in poor mechanical properties of the membranes, indicating a trade-off between the ionic conductivity and mechanical properties. Gong et al. demonstrated that anion-exchange NF composite membranes absorb more water and swell less than conventional solution-casted membranes due to aggregation of the absorbed water molecules along the fiber surface rather than having a uniform distribution across the membrane [118]. Moreover, the ion-conductive NFs offer efficient ion transport pathways by constructing continuous ion-conducting domains resulting from the molecular orientation or confined polymer chains inside the NFs [119,120,121]. Consequently, the ionic conductivity of NF composite membranes is higher than the corresponding cast membranes [122]. 

#### 3.4.1. Nanofiber Composite AEMs 

Anion-exchange (anion-conductive) NFs are applied in composite AEMs to improve the fuel cell performance. The NF network with densely cationized surfaces not only provides reliable mechanical support but also significantly improves the ionic conductivity of the composite membrane. Gong et al. used imidazolium-functionalized PSf for preparing both the electrospun NFs and matrix for composite AEMs [118]. Compared to the corresponding cast membranes, the composite membrane exhibited a significantly higher hydroxide conductivity (1.7-fold in 20 °C water and 100-fold at 60 °C, 40% RH) and improved alkaline and mechanical stabilities. Wang et al. prepared hydroxide-conductive NFs of poly(aryl ether sulfone) (PAES) with a hexa-alkyl guanidinium group side chain. The composite membranes were fabricated by immersing the NF membrane in an aqueous solution of vinyl benzyl trimethyl ammonium chloride (VBTC) and *N*,*N*’-methylene bis(acrylamide), followed by polymerization [123]. The composite membranes with NFs showed improved membrane stability and decreased water uptake compared to the membranes without NFs. Moreover, there was significant improvement in hydroxide ion conductivity. Watanabe et al. reported excellent properties of anion-conductive NFs of quaternized PAES [124]. The conductivity of the NFs (259 mS·cm^−1^) was much higher than the corresponding casted membrane (40 mS·cm^−1^) at 30 °C. The composite membranes exhibited improved membrane stability, gas permeability, and suppressed hydration swelling, thereby suggesting potential application in next-generation fuel cells. Abouzari-lotf et al. produced AEMs with polycationic side chains by radiation-induced emulsion graft copolymerization of vinylbenzyl chloride onto syndiotactic poly(propylene) and nylon-66 NF membranes, followed by crosslinking with 1,8-octanediamine and quarternizing with trimethylamine [125]. The prepared membranes exhibited adjustable IEC (1.6–2.1 mmol·g^−1^), low methanol permeability, excellent alkaline stability, and high hydroxide ion conductivity (132 mS·cm^−1^ at 80 °C). A Pt-catalyzed fuel cell using the NF composite membranes showed a peak power density of above 120 mW·cm^−2^ at 80 °C under 90% RH. Liu et al. prepared composite membranes of quaternized chitosan (QCS) and quaternized silica-coated PVDF (QSiO_2_@PVDF) electrospun NFs. The composite membranes exhibited a high ionic conductivity of 41 mS·cm^−1^ at 80 °C. Single alkaline fuel cells using the membrane demonstrated a maximum power density of 98.7 mW·cm^−2^ [126]. 

Park et al. produced composite AEMs by dual electrospinning of chloromethylated PSf and poly(phenylsulfone) (PPSU) [127]. The dual-NF membrane was quaternized with trimethylamine and then selective softening of PPSU filled the void space between the quaternized PSf fibers to obtain a dense and defect-free NF composite membrane. The composite membrane had an IEC of 1.56 mmol·g^−1^, with equilibrium swelling (93%) and desirable alkaline fuel cell hydroxide conductivity (40 mS·cm^−1^ at 23 °C in water). Similarly, new types of composite membranes of chloromethylated PSf and PPSU were prepared by partial cross-linking of chloromethylated PSf fibers with an aliphatic diamine [128] and hexanedial [129]. The remaining chloromethyl groups were finally quaternized with trimethylamine. The composite membranes exhibited an adjustable IEC, excellent hydroxide ion conductivity, and moderate water swelling. Park et al. fabricated composite AEMs by dual electrospinning of brominated PPO and PPSU [130]. The cross-linking, compression, and functionalization of as-spun fiber membranes achieved an interconnected fiber network of PPO-based polyelectrolytes with either benzyl trimethyl ammonium or 1,2-dimethyl imidazolium fixed-charge groups embedded in a PPSU matrix. The composite membranes with fixed charges of benzyl trimethyl ammonium yield a superior hydroxide conductivity in liquid water (66 mS·cm^−1^ for 50 wt.% poly(phenyl sulfone) at an IEC of 2 mmol·g^−1^). Additionally, the composite membrane exhibited reasonable water swelling (97% at room temperature), robust mechanical properties (15 MPa stress-at-break in the hydrated state), and good chemical stability in 1.0 M KOH at 60 °C, thus indicating promise for application in alkaline fuel cells. The characteristics of the reported NF composite AEMs are summarized in Table 5.

#### 3.4.2. Nanofiber Composite Proton-Exchange Membranes (PEMs)

Most commonly used PEMs in commercial fuel cells are PFSA membranes such as Gore-Select^®^ (W. L. Gore & Associates, Newark, DE, USA) and Nafion^®^. The preparation of Nafion NFs is desirable. However, it is difficult to obtain Nafion NFs through electrospinning because Nafion dispersions exhibit low solution viscosities caused by poor entanglement of polymer chains and the presence of aggregates (micelles) [131]. Therefore, it is common to add small amounts of spinning aids such as poly(*N*-vinyl pyrrolidone) [113], poly(ethylene oxide) (PEO) [132,133,134], PAA [131,135], PVA [136], PES [47], and PAN [137] to Nafion dispersions for enhancing polymer chain entanglement or suppressing aggregation formation. Subianto et al. reported that the threshold concentration of PEO for Nafion fiber formation depends on both the molecular weight of PEO and the concentration of the PFSA ionomers [134]. They found that short side chains and higher density of sulfonic acid groups in PFSA ionomers improved physical crosslinking and higher crystallinity, thus facilitating electrospinning to form Nafion fibers with a significantly lower average diameter. The proton conductivity of the composite membrane was as high as 93 mS·cm^−1^ at 120 °C and 50% RH. Choi et al. prepared proton-exchange NFs by electrospinning a blend of highly charged 825 equivalent-weight (corresponding to an IEC of 1.21 mmol·g^−1^) PFSA, PAA, and sulfonated octaphenyl polyhedral silsesquioxane [135]. After welding the intersecting NFs and compaction, the electrospun PFSA/PAA and PFSA/sulfonated octaphenyl polyhedral silsesquioxane/PAA NF membranes were impregnated with inert and hydrophobic polymer Norland optical adhesive (NOA63, Norland Products Inc., Cranbury, NJ, USA) to fill the pores between the fibers. The proton conductivity of the prepared composite membrane (83 mS·cm^−1^) was 2.4 times higher than that of the PFSA/PAA NF composite membrane (34 mS·cm^−1^) and three times higher than Nafion 212 (28 mS·cm^−1^) under the same conditions of 80 °C and 50% RH. 

**Figure 8:**
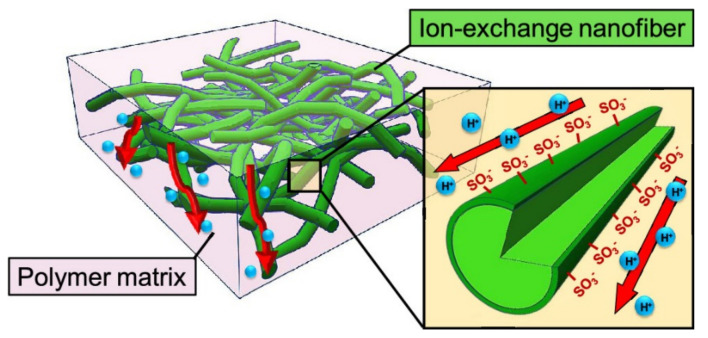
Schematic of NF composite CEMs containing a random network of cation-exchange NFs [35].

Another way to prepare NF composite PEMs is to combine a Nafion matrix with cation-exchange NFs. Cation-exchange NF networks are applied in the construction of high-performance composite PEMs. The interconnected structures and high surface area of NF networks allow for the arranged conduction groups to form affluent and long-range pathways for proton transport. Seino et al. prepared composite membranes of Nafion and PVA-based cation-exchange NFs. They found that a high density of ion-exchange groups on the NF surface improved the ionic conductivity of membranes. In other words, the three-dimensional (3D) randomly oriented proton-exchange NF networks enabled the formation of continuous ionic transport pathways in the polymer matrix (Figure 8) [35]. Wakiya et al. fabricated composite membranes composed of phytic acid-doped poly(benzimidazole) NFs in a Nafion matrix. Proton conductive pathways consisting of phosphoric acid and sulfonic acid groups formed at the NF/matrix interface. The composite membrane with a higher NF content showed higher proton conductivity and lower activation energy [138]. Li et al. used poly(styrene sulfonic acid)-modified electrospun PVDF NFs as reinforcement for the formation of Nafion-based composite membranes [139]. The polyelectrolyte induced aggregation of protogenic groups in Nafion on the NF surface to form a proton-conducting path along the NF longitudinal surface. The prepared composite membrane showed a 2.5-fold higher proton conductivity than the recasted Nafion membrane. Xu et al. prepared sulfonated PES NFs containing a porous organic cage of CC3 by solution blowing and filled the inter-fiber voids with Nafion to fabricate NF composite membranes for PEMs. The prepared membrane exhibited high proton conductivity (315 mS·cm^−1^ at 80 °C and 100% RH), low methanol permeability (0.69 × 10^−7^ cm^2^·s^−1^), excellent water absorption, thermal and dimensional stability, and single-cell performance [140]. 

PFSA ionomers have limitations such as low thermal stability and high production cost; thus, a significant effort is ongoing to develop aromatic hydrocarbon electrolyte polymers. Takemori et al. prepared composite membranes of randomly aligned sulfonated poly(imide) (PI) NFs in a sulfonated PI matrix. The proton conductivity of the composite membrane was higher than the conventional solution-casted membrane [115]. Moreover, the membrane stability significantly improved with the increase in the NF content. Tamura et al. prepared composite membranes of uniaxially aligned ultrafine sulfonated PI NFs and a sulfonated PI matrix [141]. The proton conductivity of the composite membranes in the parallel direction to the nanofiber alignment increased with decreasing NF diameters, reaching 300 mS·cm^−1^ (90 °C and 98% RH) with a diameter of 77 nm. Conversely, the proton conductivity of the composite membranes in the perpendicular direction to the aligned fiber was similar to that of the membrane without NFs. Choi et al. developed a composite membrane of a 3-D interconnected network of electrospun sulfonated PAES NFs filled with an inert/uncharged polymer (UV-cured NOA 63, a solvent-less photo-curable urethane-based pre-polymer) [142]. The proton conductivity of the composite membranes linearly increased with fiber volume fraction and properties of the NF composite membranes were comparable to those of commercial Nafion 117. They also attempted to enhance the proton conductivity by adding sulfonated polyhedral oligomeric silsesquioxane to the sulfonated PAES to fabricate NFs. The prepared composite membrane exhibited a proton conductivity that is 2.4 times higher than Nafion 212 under the same conditions [143]. Wu et al. used oriented electrospun NF membranes of sulfonated poly(phthalazinone ether sulfone ketone) and metal-organic frameworks (MOFs) as PEMs at high temperatures under anhydrous conditions [144]. The proton conductivity reached 82 mS·cm^−1^ at 160 °C under anhydrous conditions for the highly oriented nanofibers with a methanol permeability of ~6% of Nafion-115. Ibaraki et al. fabricated polymer electrolyte NF composite membranes by embedding phytic acid-doped poly(benzimidazole) NFs in sulfonated PI. The phosphoric acid groups in the doped acid and sulfonic acid groups of the polymer electrolyte matrix aggregated at the NF surface, forming an effective proton-conducting pathway. The functionalized NF composite membranes exhibited a high through-plane conductivity of 161 mS·cm^−1^ at 80 °C and 100% RH [145]. Table 6 summarizes the characteristics of the reported NF composite PEMs.

#### 3.4.3. NF Composite IEMs Containing Acid-Base Pairs 

An alternative approach to construct ion conduction paths through NF composite IEMs is to form acid-base pairs at the NF/matrix interface. The acid-base pairs such as -SO_3_H and -NH_2_ formed at the NF/matrix interface provide abundant hydrogen-bonded networks for proton hopping. The most reported NF composite IEMs are based on the NFs containing -NH_2_ groups and the Nafion matrix. The -NH_2_ groups in NFs are considered as effective proton sites offering tailored hydrogen-bonding networks within the acid matrix, providing for efficient proton transport. Sood et al. prepared composite membranes by incorporating electrospun NFs of PSf functionalized with 4-heptyl-1,2,3-triazole (PSUT) into a short-side-chain-type PFSA matrix (Aquivion^®^, Solvay S. A., Brussels, Belgium). The membrane-electrode assemblies incorporating the composite membrane exhibited five times superior durability compared to the pristine Aquivion membrane [146]. Wang et al. prepared a hybrid PEM by incorporating an electrospun hybrid MOF (UiO-66-NH_2_)-anchored-NF framework into a Nafion matrix. The -SO_3_H groups from Nafion and -NH_2_ groups from UiO-66-NH_2_ formed acid-base pairs, acting as proton-conducting highways. The prepared membrane exhibited improved proton conductivity. Meanwhile, the good compatibility of the NF framework with the Nafion matrix, due to tight electrostatic interactions between -NH_2_ and -SO_3_H groups, endowed the hybrid membrane with a suppressed methanol permeability of 7.54 × 10^−7^ cm^2^·s^−1^ [147]. Zhao et al. proposed a strategy for constructing ion-nanochannels by combining a zeolitic imidazolate framework (ZIF-8) with a 3D network structure of poly(m-phenylene isophthalamide) (PMIA) NFs via a hydrothermal method. The ZIF-8 on the PMIA NFs generated longer and consecutive channels in the composite membrane due to the acid-base pairing between SO_3_H (Nafion) and N-H (imidazole molecule of ZIF-8) [148]. 

In contrast, the composite membranes incorporating the NFs with acid groups into the chitosan matrix were also reported. Gong et al. coated phosphotungstic acid, a strong heteropolyacid and a super proton conductor, on the surface of PVDF NFs and filled the NF network with chitosan to prepare a composite membrane [149]. The proton conductivity of the prepared composite membrane was approximately one order of magnitude higher than chitosan-filled pure PVDF membrane. Zhang et al. used sPEEK to prepare NF membranes and a composite with the matrix of chitosan mixed with sulfonated halloysite nanotubes [150]. The prepared NF composite membranes contain dual-interfacial proton-conducting pathways at the sPEEK/chitosan and chitosan/sulfonated halloysite nanotube interfaces, leading to ultrafast proton conduction under both hydrated and anhydrous conditions. Moreover, Li et al. fabricated hybrid sPEEK NFs membranes with uniformly distributed 2–5 nm quantum dots (QDs) and then incorporated it in the chitosan matrix to prepare composite membranes. The QDs provide a large number of proton-conducting groups (-NH-/-NH_2_ and -COOH) and the -NH-/-NH_2_ groups form ordered acid-base pairs with the -SO_3_H groups in sPEEK, providing low-barrier pathways for proton hopping through the NF. The prepared membrane displayed significantly enhanced through-plane and in-plane proton conduction and decreased transfer anisotropy. The through-plane conductivity was as high as 456 mS·cm^−1^ at 90 °C and 100% RH, almost three times that of the membrane without QDs [151]. 

Amino-acid-functionalized NFs are also used for the preparation of NF composite IEMs containing acid-base pairs [152,153,154]. Wang et al. fabricated composite membranes of amino-acid-functionalized SiO_2_ NFs and Nafion to investigate the different polar groups of amino acids that contribute to membrane properties. The introduction of NFs significantly improved the proton conductivity and dimensional stability of the composite membrane. The highest proton conductivity of 240 mS·cm^−1^ (80 °C, 100% RH) was achieved with the amino acid cysteine [155]. Wang et al. fabricated composite membranes by impregnating oxidized cysteine-functionalized PVDF NFs into the Nafion matrix. The composite membranes exhibited the highest proton conductivity of 220 mS·cm^−1^ (80 °C, 100% RH) and a maximum power density of 108.42 mW·cm^−2^ (60 °C, 100% RH), almost twice that of the Nafion 117 membrane [156]. Table 7 summarizes the characteristics of the reported NF composite IEMs containing acid-base pairs.

## 4. Future and Outlook

This review presents the recent applications of NF-based IEMs in catalysis, environment, and energy fields. IEX-NF membranes with a high surface area increase the availability of surface active sites/functional groups, thereby improving ion-exchange/adsorption capacity and catalytic efficiency. In addition, IEX-NF membranes improve the ion diffusion properties due to their highly porous and low-tortuous structures. Therefore, the membranes provide rapid kinetics as well as high adsorption capacity (for heavy metals, dyes, and proteins) and efficient/continuous chemical reactions (e.g., efficient water dissociation in the bipolar membrane process) in a liquid phase. These are major dimensional advantages of NFs (high-aspect-ratio 1D nanomaterial) compared with advanced 2D nanomaterials (e.g., graphene, graphene oxides, and MXenes) [157]. In addition, the 3D framework of IEX-NFs provides efficient ion conduction pathways through composite membranes, improving both the electrochemical and mechanical performances. Thus, IEX-NFs are promising materials particularly for achieving the sixth and seventh Sustainable Development Goals (SDGs) of “Clean Water and Sanitation” and “Affordable and Clean Energy”, respectively. 

There are two major barriers to the practical realization of IEX-NFs, production costs and safety [9]. The cost is mainly dependent on the production cost of NFs because the production scheme is basically the same as for the conventional IEX fibers, which are successfully commercialized. Recently, the productivity of NF production technologies increased significantly with the introduction of high-throughput production systems at the semi-industrial scale, such as multi-nozzle electrospinning, free-surface electrospinning, solution blow spinning, and centrifugal spinning [9,27,29]. We hope that the production cost will drop dramatically with increased demand and production scale-up.

Concerning safety, at present, there are no generalized regulations for the safety of nanomaterials including NFs because of their diversity and complexity. To the best of our knowledge, the toxicity of the NFs and NF non-woven membranes prepared from commonly-used synthetic and natural polymers has not been reported so far. In actuality, the length of the typical NFs is more than several centimeters and many polymer NFs have been extensively studied for biomedical uses such as tissue engineering and drug/gene delivery. The toxicity of nanomaterials depends on the individual raw materials as well as specific production methods and the forms used. For example, shape-dependent toxicity has been reported for short strands (<100 μm) [9]. We have to check the safety carefully for the commercialization of IEX-NFs.

The establishment of a high-throughput, scalable, low-cost, and safe NF production process is inevitable for the industrialization of porous IEX-NF membranes and/or dense composites of IEX-NF membranes and polymer matrices (NF-composite IEMs) [158]. In addition, rational design of high-performance IEX-NF membranes and their composites requires an in-depth understanding of microstructures based on quantitative analyses of the surface/interfacial area of NFs, connectivity of pores/NFs, and systematic studies of microstructure ion permeation/separation relationships based on the theoretical framework. Recently, Hou et al. produced NF composite membranes containing both positively and negatively charged groups by impregnating electrospun NF mats of bromomethylated poly(2,6-dimethyl-1,4-phenylene oxide) (PPO) into sulfonated PPO followed by quaternization of the bromomethyl groups. The prepared membrane exhibited superior monovalent permselectivity in ED: monovalent cations can be separated from divalent cations, which was ascribed to the different repulsion forces from the positively charged nanofibers throughout the whole membrane [159]. In general, ionic transport phenomena across charged membranes, including conventional IEMs, can be theoretically described by the Teorell, Meyer, and Sievers (TMS) theory, which is based on the Donnan equilibrium and the Nernst–Planck equation [160]. This theory assumes that the fixed-charge groups, i.e., ionizable active sites, are homogeneously distributed in the membrane. Therefore, extending the theoretical framework and/or the establishment of a new one is strongly required for the rational design of NF-based IEMs with improved ionic transport properties. We believe that such endeavors will position IEX-NFs as key building blocks for “Nanostructured Ion-Exchange Membranes” [161], with a significant expansion of their applications. 

## Figures and Tables

**Table 1:** Characteristics of conventional and nanofiber ion-exchange membranes.

	Traditional IEMs	IEX-NF Non-Woven Membranes
Membrane structure	dense	highly porous (typically ≥ 80%) [36]
Ion-exchange capacity [mmol·g^−1^]	0.6–3.5 *	0.44 [37]–5.4 [38]
Surface area [m^2^·g^−1^]	<0.01	2.5 [39]–228 [40]
Permeability	medium~high (e.g., area resistance of 1.2~12 Ω·cm^2^ *)	very high
Selectivity	high (e.g., transport number of 0.92 ~>0.99 *)	low as a membrane high as an adsorbent

* The data from [41].

**Table 2:** Recently reported anion-exchange NF membranes for adsorption.

Ref.	Material	IEX Group	NF Diameter [nm]	IEC^a^ [mmol·g^−1^] /SSA^b^ [m^2^·g^−1^]	Adsorbent (@pH)	Adsorption Capacity [mg·g^−1^]
Heavy metals						
[40]	PVA/MA/ATM	-NH_2_, -NHR	115–140	228.4 ^b^	Pd^2+^, Pt^4+^ (pH 1.1)	69 (Pd^2+^) 112 (Pt^4+^)
[68]	chitosan	-NH_2_	129	13.2 ^b^	As^5+^ (pH 3.4)	30.8
[69]	chitosan	-NH_2_	75	-	Cr^6+^ (pH 6)	20.5
[70]	chitosan/nylon-6	-NH_2_	301	-	Cu^2+^ (pH 4)	240
[71]	PAN/polypyrrole	pyrrole	258	-	Cr^6+^ (pH 2)	61.80
[72]	PAN/polyaniline	aniline	301–420	-	Cr^6+^ (pH 2)	71.28
[75]	PEI/EPI/PAN	-NH_2_, -NHR, -NR_2_	831	-	Cu^2+^, Pb^2+^ (-)	350 (Cu^2+^) 290 (Pb^2+^)
Dyes						
[74]	m-PEI/PVDF	-NH_2_, -NHR, -NR_2_	50–200	-	MO (pH 7)	633
[75]	PEI/EPI/PAN	-NH_2_, -NHR, -NR_2_	831	-	MO (-)	637
[76]	PDA/DCA-COOH	-NH_2_	-	-	MB, CR (pH 11)	69.9 (MB) 67.30 (CR)
Proteins						
[38]	chitosan	-NH_2_	~100	5.4 ^a^/25.9 ^b^	DNA (pH 4)	600
[77]	cellulose/PEAA	-NH_2_, -NHR	550	-	BSA (pH 8)	239
[78]	PAN-GMA-DEA PSf-GMA-DEA	-NR_2_	~400 ~2500	-	BSA (pH 7)	~100 mg/mL ~200 mg/mL
[79]	cellulose/diethylaminoethyl ligand	-NR_2_	Tens of nanometers to microns	-	BSA (pH 8)	40.0
[81]	PAN/Tris	-NH_2_	-	-	Lys (pH 7)	1362

^a^ ion-exchange capacity; ^b^ specific surface area. Abbreviations: PAN, poly(acrylonitrile); MA, maleic anhydride; ATM, acryloyl thioamide monomer; PVA, poly(vinyl alcohol); m-PEI, methacrylate polyethylenimine; PVDF, poly(vinylidene fluoride); EPI, epichlorohydrin; PDA:DCA, poly(dopamine): deacetylated; PEAA, polyethyleneamidoamine; GMA, glycidyl methacrylate; DEA, diethylamine; PSf, poly(sulfone); Tris, tris(hydroxymethyl)aminomethane; MO, methyl orange; MB, methylene blue; CR, congo red; BSA, bovine serum albumin; and Lys, lysozyme.

**Table 3:** Recently reported cation-exchange NF membranes for adsorption.

Ref.	Material	IEX Group	NF Diameter [nm]	IEC^a^ [mmol·g^−1^] /SSA^b^ [m^2^·g^−1^]	Adsorbent (@pH)	Adsorption Capacity [mg·g^−1^]
Heavy metals						
[82]	PFSA/PVP	-SO_3_H	377	1.07 ^a^	Cu^2+^, Ca^2+^ (pH 5)	43.10 (Cu^2+^) 22.37 (Ca^2+^)
[84]	PS/SBS	-SO_3_H	1000	3.08 ^a^	Cu^2+^ (pH 4)	3.08 mmol·g^−1^
[85]	sPES	-SO_3_H	1170	1.6 ^a^	NH_4_^+^ (pH 7)	14.08
[86]	PVA/PAA-PO_4_	-H_2_PO_4_	278	-	Uranium	277.78
[89]	cellulose/PIA	-COOH	200–500		Cd^2+^ (-)	222
[92]	cellulose/thiol	-SH	418	-	Cu^2+^, Cd^2+^, Pb^2+^ (pH 4)	Cu^2+^(49.0), Cd^2+^(45.9), Pb^2+^(22.0)
Dyes						
[94]	sPES	-SO_3_H	62	-	MB (pH 6.8)	6.6
[95]	PES/P(AA-MMA)	-COOH	503	-	MB (pH 9)	2258
[96]	SA/CaCl_2_	-COOH	155	13.56	MB (pH 6)	2230
Proteins						
[37]	PAN	-COOH	200–250	0.44 ^a^/6.18 ^b^	Lys (pH 9)	83.2
[39]	EvOH/CCA	-COOH	562	2.52 ^b^	Lys (pH 6)	284
[97]	cellulose/PAA	-COOH	~500	-	Lys (pH 7)	2.6
[98]	PVA/MAH	-COOH	226	3.2 ^b^	Lys (pH 6)	177

^a^ ion-exchange capacity; ^b^ specific surface area. Abbreviations: PIA, poly(itaconic acid); PS, poly(styrene); SBS, styrene-butadiene-styrene block copolymer; PFSA, perfluorinated sulfonic acid; PVP, poly(N-vinyl pyrrolidone); sPES, sulfonated poly(ether sulfone); PVA, poly(vinyl alcohol); PAA, poly(acrylic acid); PES, poly(ether sulfone); P(AA-MMA), acrylic acid and methyl methacrylate copolymer; MAH, maleic anhydride; EvOH, ethylene-vinyl alcohol; CCA, citric acid; PAN, poly(acrylonitrile); MB, methylene blue; and Lys, lysozyme.

**Table 4:** Recently reported hybrid ion-exchange NF membranes for adsorption.

Ref.	Material	IEX Site/ IEXgruoup	NF Diameter [nm]	SSA [m^2^·g^−1^]	Adsorbent (@pH)	Adsorption Capacity [mg·g^−1^]
Heavy metals						
[101]	PAN/α-Fe_2_O_3_	α-Fe_2_O_3_	~200	-	Pb^2+^ (pH 4.8)	81.97
[102]	PEI-AN/iron oxide	Fe_2_O_3_	230	4.347	Ni^2+^ (pH 8)	102
[103]	PVA/iron oxide	Fe_3_O_4_	120	-	As^5+^ (pH 3)	52
[104]	PVDF/TBAC-MnO_2_	MnO_2_	-		Pb^2+^ (pH 6)	318.47
[105]	PVA/zeolite nanoparticle	zeolite	170	212	Cd^2+^, Ni^2+^ (pH 5)	838.7 (Cd^2+^) 342.8 (Ni^2+^)
[106]	CA/HAp	phosphate	120	-	Fe^3+^, Pb^2+^ (pH 6)	45.45 (Fe^3+^) 49.75 (Pb^2+^)
[107]	CA/zeolite nanoparticle	zeolite	139	-	Cu^2+^, Pb^2+^ (pH 6.6)	1.22 mmol·g^−1^ (Cu^2+^) 1.1 mmol·g^−1^ (Pb^2+^)
[108]	PET/wool keratin	-NH_2_	610	-	Cr^6+^ (pH 3)	75.86
[110]	Chitosan/lanthanum	-NH_2_, La(OH)_3_	130–310	-	Arsenate (pH 6)	83.6
[111]	APAN/AC	-NHR, -NH_2_	256	76.2	Cr^6+^	284
Dyes						
[109]	PAA/SiO_2_	-COOH	300–700	212	MG	220.49
[111]	APAN/AC	-NHR, -NH_2_	256	76.2	MO (pH 3)	248

Abbreviations: PAN, poly(acrylonitrile); PEI-AN, poly(etherimide-acrylonitrile); PVA, poly(vinyl alcohol); CA, cellulose acetate; PVDF, poly(vinylidene fluoride); TBAC, tetrabutylammonium chloride; PET, poly(ethylene terephthalate); Hap, hydroxyapatite; APAN, amine-modified PAN; AC, activated carbon; SA, sodium alginate; PAA, poly(acrylic acid); MO, methyl orange; MG, malachite green.

**Table 5:** Recently reported nanofiber composite anion-exchange membranes for fuel cells.

Ref.	NF			Matrix	Composite Membrane				
	Material (IEX Group)	Diameter [nm]	IEC [mmol·g^−1^]	Material (IEX Group)	IEC [mmol·g^−1^]	Thickness [µm]	NF Content (%)	Water Uptake [%]	Hydroxide Conductivity ^a^ [mS·cm^−1^] (°C)
[118]	IM-PSF (imidazolium)	156	1.78	IM-PSF (imidazolium)	1.78	100	58.5 (wt.)	250 (40.6 ^b^)	70.2 (60)
[123]	PES-G-Cl (guanidinim TMA)	80–100	-	VBTC/MBA (TMA)		-	-	20.1 (10.1 ^b^)	92 (70)
[124]	Q-PAES (-N^+^H_3_)	142	1.51	Q-PAES (-N^+^H_3_)	1.51	-	20 (wt.)	-	83 (30)
[125]	*syn*-PP nylon-66 (TMA)	335 (syn-PP) 90 (nylon-66)	1.9–2.1	-	1.7–2.1	15	-	-(32 ^b^)	132 (80)
[126]	QSiO_2_@PVDF (TMA)	-	0.60	QCS (-N^+^H_3_)	-	-	-	130–150	41 (80)
[127]	CM-PSF (TMA)	950	2.02–2.47	PPSU (-)	1.27–1.56	-	63 (wt.)	93	40 (23)
[128]	diamine crosslinked CM-PSF (TMA)	700	3.1	PPSU (-)	2.01	-	65 (wt.)	144	65 (23)
[129]	diol crosslinked CM-PSF (TMA)	814	2.8	PPSU (-)	1.99	-	65 (wt.)	136	57 (23)
[130]	BrPPO (TMA)	400	4.0	PPSU (-)	1.2–2.8	40	50 (wt.)	96	66 (23)

^a^ meaured in water. Hydroxide conductivity is much higher than chloride conductivity. ^b^ swelling ratio. Abbreviations: IM-PSF, imidazolium-functionalized poly(sulfone); VTBC, (vinylbenzyl) trimethylammonium chloride; MBA, N,N’-methylene bis(acrylamide); PES-G-Cl, poly(aryl ether sulfone) with hexaalkkyl guanidinium group side chain; Q-PAES, quaternized-poly(arylene ether sulfone); syn-PP, syndiotactic poly(propylene); QCS, quaternized chitosan; QSiO_2_, quaternized silica; PVDF, poly(vinylidene fluoride); PPSU, poly(phenylsulfone); CM-PSF, chloromethylated poly(sulfone); BrPPO, brominated poly(phenylene oxide); and TMA, trimethylamine.

**Table 6:** Recently reported nanofiber composite proton-exchange membranes for fuel cells.

Ref.	NF			Matrix	Cpmposite Membrane				
	Material (IEX Group)	Diameter [nm]	IEC [mmol·g^−1^]	Material (IEX Group)	IEC [mmol·g^−1^]	Thickness [µm]	NF Content (%)	Water Uptake [%]	Proton Conductivity [mS·cm^−1^] (°C,%RH)
[132]	PFSA/PEO (-SO_3_H)	162	-	NOA63 (-)	-	90–120	75 (vol.)	52	160 (80, 80)
[135]	PFSA/sPOSS/PAA (-SO_3_H)	275	2.4	NOA63 (-)	-	-	74 (vol.)	32	21 (120, 20) 107 (120, 50) 498 (120, 90)
[35]	PVA-b-PSS (-SO_3_H)	264	0.46	Nafion (-SO_3_H)	1.02	23	15 (wt.)	44	63(25,-)
[139]	PSSA-PVDF (-SO_3_H)	300	-	Nafion (-SO_3_H)	-	100	10 (wt.)	36	106 (95,-)
[140]	sPES/POC (-SO_3_H)	236	-	Nafion (-SO_3_H)	0.90	110	10 (wt.)	-	315 (80, 100)
[115]	sPI (-SO_3_H)	208	1.5	sPI (-SO_3_H)	2.7	50	20 (wt.)	53	330 (80, 98)
[141]	sPI (-SO_3_H)	77	-	sPI (-SO_3_H)	1.63	30	30 (wt.)	38	300 (90, 98) (parallel)
[142]	sPAES (-SO_3_H)	110	2.5	NOA63 (-)	-	39	60 (vol.)	-	90 (25, -)
[143]	sPAES/sPOSS (-SO_3_H)	491	3.2	NOA63 (-)	-	70	70 (vol.)	-	94 (30, 80)
[144]	sPPESK/MOFs (-SO_3_H)	200	-	-	-	45	-	-	82 (160, 0)
[145]	Phy /PBI (-H_2_PO_4_)	162	-	sPI (-SO_3_H)	3.2	32	10 (wt)	19	161 (80,100)

Abbreviations: NOA, Norland optical adhesive; PFSA, perfluorinated sulfonic acid; PEO, poly(ethylene oxide); sPOSS, sulfonated octaphenyl polyhedral silsesquioxane; PAA, poly(acrylic acid); PVA-b-PSS, poly(vinyl alcohol-b-styrene sulfonic acid); PSSA, poly(styrenesulfonic acid); PVDF, poly(vinylidene fluoride); sPES, sulfonated poly(ethersulfone); POC, porous organic cage; sPI, sulfonated poly(imide); sPAES, sulfonated poly(arylene ether sulfone); sPPESK, sulfonated poly(phthalazinone ether sulfone ketone); MOF, metal-organic framework; Phy, phytic acid; and PBI, poly(benzimidazole).

**Table 7:** Recently reported NF composite IEMs containing acid-base pairs for fuel cells.

Ref.	NF			Matrix	Compospite Membrane				
	Material (IEX Group)	Diameter [nm]	IEC [mmol·g^−1^]	Material (IEX Group)	IEC [mmol·g^−1^]	Thickness [µm]	NF Content (%)	Water Uptake [%]	Proton Conductivity [mS·cm^−1^] (°C,%RH)
[147]	MOF/sPES (-NH_2_, -SO_3_H)	180	-	Nafion (-SO_3_H)	1.1	70	40 (wt.)	37	270 (80, 100)
[148]	ZIF/PMIA (imidazole)	230	-	Nafion (-SO_3_H)	1.13	50–70	15 (wt.)	-	258 (80, 100)
[150]	sPEEK (-SO_3_H)	140	-	CS/SHNTs (-SO_3_H, -NH_2_)	0.75	43–58	-	70.5	117.7 (90, 100) 19.95 (100, 0)
[151]	sPEEK quantum dots (-NH-, -NH_2_, -SO_3_H)	-	0.60	Chitosan (-NH_2_)	0.47	-	-	85	456 (90,100)
[152]	L-lysine/PAN (-NH_2_, -NHR)	100–300	-	Nafion (-SO_3_H)	1.13	40–70	10 (wt.)	54.4	263 (80, 100)
[153]	γ-PGA/PLA (-COOH, -NHR)	659	-	sPES (-SO_3_H)	-	200	30 (wt.)	-	261 (80, 100)
[154]	L-Argnine/PAN (-COOH, -NHR, -NH_2_)	100–300	-	sPSF (-SO_3_H)	1.24	50–80	5 (wt.)	61	216(80, 100)
[155]	Cysteine/SiO_2_ (-COOH, -SH, -NHR)	250–500	-	Nafion (-SO_3_H)	-	-	10 (wt.)	-	242 (80, 100)
[156]	Cysteine/PVDF (-COOH, -NHR, -SO_3_H)	40–220	-	Nafion (-SO_3_H)	1.31	120	30 (wt.)	62.1	220 (80, 100)

Abbreviations: MOF, metal-organic framework; sPES, sulfonated poly(ether sulfone); sPEEK, sulfonated poly(ether ether ketone); ZIF, zeolitic imidazolate framework; PMIA, poly(m-phenylene isophthalamide); CS, chitosan; SHNT, fulfonated halloysite nanotube; PAN, poly(acrylonitrile); PVDF, poly(vinylidene fluoride); γPGA, γ-poly(glutamic acid); PLA, poly(lactic acid); and sPSF, sulfonated poly(sulfone).
